# Computational Growth Modeling: Assessing Longitudinal Change with Joint Models

**DOI:** 10.21203/rs.3.rs-11269872/v1

**Published:** 2026-10-21

**Authors:** Jeremy Haynes, Nathaniel Haines, Holly Sullivan-Toole, Thomas Olino

## Abstract

Computational models involve fitting a behavioral model to data from a behavioral task to estimate parameters characterizing task performance. Frequently, computational models are used to clarify associations between psychological constructs (e.g., reward learning) and mental illness (e.g., depression). A common method for assessing the etiology of mental illness involves longitudinal research designs that evaluate how behavioral processes change over time. In this paper, we illustrate how hierarchical computational modeling can be extended to longitudinal data via joint modeling. We first provide a brief overview of common approaches to longitudinal modeling and then describe how to integrate computational and longitudinal analyses using joint modeling; an approach we refer to as *computational growth modeling* . Next, using simulation, we show how computational growth modeling improves upon more traditional approaches for analyzing longitudinal change in parameters from a simple reinforcement learning model. In particular, the computational growth modeling approach estimates person-level longitudinal growth parameters better than a more traditional, two-stage approach whereby computational parameters and growth trajectories are modeled separately. After this initial proof-of-concept simulation, we show how computational growth modeling can be applied to real longitudinal data collected from five waves of adults tested on the play-or-pass Iowa Gambling Task. Ultimately, our goal is to illustrate how to use the computational growth modeling approach, which shows promise for more precisely modeling changes in behavioral processes across time, and has particular relevance to understanding how the development of these behavioral processes may play an etiological role in psychopathology.

